# Increased Risk for Thromboembolism After Fontan Surgery: Considerations for Thromboprophylaxis

**DOI:** 10.3389/fped.2022.803408

**Published:** 2022-03-28

**Authors:** Suelyn Van Den Helm, Christopher Noel Sparks, Vera Ignjatovic, Paul Monagle, Chantal Attard

**Affiliations:** ^1^Haematology Research, Murdoch Children’s Research Institute, Melbourne, VIC, Australia; ^2^Department of Paediatrics, The University of Melbourne, Melbourne, VIC, Australia; ^3^Department of Anatomy and Physiology, The University of Melbourne, Melbourne, VIC, Australia; ^4^Kids Cancer Centre, Sydney Children’s Hospital, Randwick, NSW, Australia; ^5^Department of Haematology, Royal Children’s Hospital, Melbourne, VIC, Australia

**Keywords:** Fontan, anticoagualtion, direct-acting oral anticoagulant (DOAC), warfarin, aspririn, stroke, thromboemboilc disease

## Abstract

The Fontan circulation introduces an increased risk of thromboembolism which is associated with substantial mortality and morbidity. Adverse outcomes of thromboembolic complications post-Fontan surgery vary in both nature and severity, ranging from local tissue infarction and pulmonary embolism to Fontan failure and ischemic stroke. Furthermore, recent studies have identified that subclinical stroke is common yet underdiagnosed in Fontan patients. Fontan patients are commonly treated with antiplatelet agents and/or anticoagulants as primary thromboprophylaxis. Optimal thromboprophylaxis management in the Fontan population is still unclear, and clinical consensus remains elusive despite the growing literature on the subject. This perspective will describe the nature of thromboembolism post-Fontan surgery and provide evidence for the use of both current and emerging thromboprophylaxis options for children and adults living with Fontan circulation.

## Introduction

The Fontan procedure has enabled increasing numbers of pediatric patients with an array of single-ventricle physiologies to survive into adulthood. However, the Fontan circulation introduces an increased risk of thromboembolism (TE), which is in turn associated with substantial mortality and morbidity (1–3). Adverse outcomes of thromboembolic complications post-Fontan surgery vary in both nature and severity, ranging from local tissue infarction and pulmonary embolism, to Fontan failure and ischemic stroke (2, 4). Fontan patients are therefore commonly treated with antiplatelet agents or various anticoagulants, as prophylaxis to attenuate this increased thrombotic risk. However, the optimal management in the Fontan population remains unclear, and clinical consensus regarding thromboprophylaxis remains elusive (5).

This perspective aims to provide a brief outline of the nature of TE post-Fontan surgery, as well as insight into the growing body of literature evaluating the outcomes of conventional, as well as emerging thromboprophylactic regimes in the pediatric and adult Fontan populations.

## TE Post-Fontan Surgery

Post-Fontan TE in the short- and long-term following surgery remains a significant complication of the procedure, and an enduring cause of morbidity and mortality (6, 7). Thromboembolic complications arising from the Fontan circuit commonly present in the arterial circulation as intracardiac thrombosis, ischemic stroke or arterial embolism, and in the venous circulation as central venous thrombosis or pulmonary embolism (2–4, 8, 9).

**FIGURE 1:**
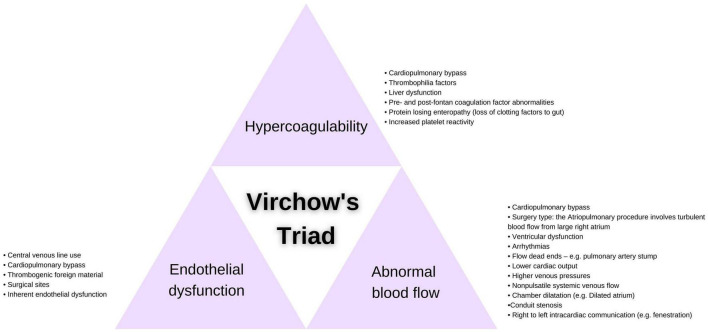
Contributors to Virchow’s triad present in Fontan patients.

The Fontan circulation itself presents a highly thrombogenic environment and an accompanying complex pathophysiology, impacting all three elements of Virchow’s triad: endothelial cell dysfunction, abnormal blood flow and a hypercoagulable state (10) (Figure 1).

Endothelial injury results directly from surgical disruption of the endothelium and the introduction of thrombogenic prosthetic material in the Fontan circuit (11, 12). As a consequence of endothelial cell activation, release of anti-thrombotic factors is impaired, and secretion of pro-thrombotic mediators is enhanced, priming the Fontan vasculature for thrombus formation (13–15).

Abnormal blood flow in the Fontan circulation is caused by turbulence, stasis and a reduced pulmonary artery flow rate dictated by the low returning venous pressure (16), all of which contribute to local initiation of coagulation and an increased thrombotic risk (17).

Hypercoagulability caused by coagulation factor abnormalities has been outlined in a number of studies both prior to and following the Fontan procedure (11, 18–20). In particular, decreased levels of circulating anticoagulants Protein C, Protein S and antithrombin have been reported (17, 19–21), though a lack of adequate pediatric reference ranges has limited meaningful interpretation of these reports (2, 10). An increased level of coagulation factor VIII and decreased levels of factors II, V, VII and X in Fontan patients have also been described when compared to age-matched controls (22). Coagulation abnormalities being compounded by the development of liver dysfunction and/or protein-losing enteropathy post-Fontan surgery (and its associated serum protein imbalances) presents an additional mechanism for the dysregulation of hemostasis (23–25). There is a paucity of pediatric cohort studies using age-appropriate reference intervals to investigate thromboembolic phenomena in the Fontan circulation, limiting insight into coagulopathy for the youngest post-Fontan patients.

Cardiac surgery increases the likelihood of post-operative thromboembolic complications in both children and adults (4, 26–28). The reported increased risk of thrombosis following Fontan surgery is likely multi-factorial, owing to the insertion of central venous lines, the use of cardiopulmonary bypass and the variable surgical manipulation of tissue (29–31). Recent studies have also suggested that the altered hemodynamics resulting from stenosis and thrombosis may be related to the graft material selected and the Fontan procedure performed (12), with Deshaies et al. specifying a possible lower thromboembolic risk in the case of extracardiac conduit Fontan surgery (32). However, a number studies have found no correlation between Fontan circuit type and thrombosis (7, 25), and further research is needed to investigate these conflicting findings.

Despite TE being recognized as a common complication post-Fontan surgery, there is considerable variation in the reported incidence. For example, the incidence of venous thrombosis varies from 4 to 19% and the incidence of stroke ranges from 3 to 19% (33–37). Additionally, the incidence of intracardiac thrombosis has been reported to be between 17 and 33% (38–40), with the thrombi most commonly observed in the systemic venous atrium (48%) and pulmonary venous chamber (44%)(25).

A prospective, multi-center, randomized controlled trial conducted by Monagle et al. assessed thromboprophylaxis post-Fontan surgery (41) and demonstrated a clinically detected incidence of thrombosis of 7% and an overall incidence of thrombosis within the first two years post-Fontan surgery of 22%. Interestingly, all TEs observed in this study were venous and there was no record of arterial thrombosis. A *post hoc* analysis of Monagle et al.’s trial by McCrindle et al. demonstrated a time-related freedom from thrombosis of 69% at 2.5 years post-randomization (31). The highest time-related risk of thrombosis was observed during the immediate post-operative period up until six months post-surgery which was followed by a lower but increasing risk of thrombosis in the next two years. These results are supported by multiple studies which have also observed an immediate high risk of thrombosis in the first year post-Fontan (37, 39, 42–44) with thrombosis risk leveling off at 3.5 years before being followed by a second peak at 10 years post-Fontan (34, 45). Regarding long term incidence of TE, an Australian national registry including 1006 survivors of the Fontan operation demonstrated freedom from thromboembolic events was 82% at 25 years post-Fontan (95% CI, 74–87%) (46).

Interestingly there is emerging evidence that subclinical thrombosis is highly prevalent following Fontan surgery. A recent cross-sectional study that utilized magnetic resonance imaging (MRI), demonstrated that stroke was highly prevalent with an incidence of 39% at > 5 years post Fontan (3). Of these, only 6% of patients were diagnosed clinically. Similarly, Monagle et al. determined that the overall thrombosis rate was 22%, with a clinically detected thrombosis rate of 7%, suggesting that silent thrombosis is highly prevalent in this population (41). Whether silent thrombosis is clinically important remains unknown, however, given the widespread nature, certainly warrants further investigation.

Discrepancies in reports of thromboembolic complications can likely be attributed to variability in outcomes measured (e.g., intracardiac thrombus, pulmonary embolism, clinical or silent thrombosis), diagnostic imaging technique used, patient age, thromboprophylactic regimen, and inconsistencies in follow-up duration (1, 5, 7, 9, 47–49). Additional prospective studies are therefore necessary to reconcile these differences.

## Thromboprophylaxis in the Fontan Population

The current clinical guidelines and consensus denote that thromboprophylaxis post-Fontan surgery is appropriate to reduce the risk of thrombosis in the Fontan circulation. Guidelines from the American Heart Association (AHA), American College of Cardiology (ACC) and the American College of Chest Physicians (ACCP) all nevertheless highlight that additional studies are needed to provide sufficient evidence for defining an optimal post-Fontan antithrombotic regime (50–53). The latest AHA recommendations state:

1. “Given the increased risk of the Fontan population for thromboembolic events, some form of thromboprophylaxis is warranted.
2. It is reasonable for all patients with a Fontan-class circulation to receive aspirin as thromboprophylaxis, whereas anticoagulation with warfarin should be reserved for patients with presumed risk factors, previous thrombotic events, or for older Fontan populations.
3. Both antiplatelet and anticoagulant agents retain a significant residual risk of post-Fontan surgery thrombosis.
4. The comparative safety and efficacy of DOACs for thromboprophylaxis in the Fontan patient remains to be determined.”

For children post Fontan surgery, the ACCP recommends aspirin or therapeutic UFH followed by vitamin K antagonists such as warfarin. The ACCP guidelines state that aspirin use for pediatric antiplatelet therapy should be administered in doses of 1–5 mg/kg per day, and pediatric patients receiving VKAs should be monitored to achieve a target INR of 2.5 (range 2.0–3.0) (51). The optimal duration of thromboprophylaxis remains to be determined however many patients continue therapy for life.

The emerging use of direct oral anticoagulant (DOAC) therapies post-Fontan surgery may present a promising alternate course of thromboprophylaxis in both children and adults. Studies of emerging anticoagulants must be carefully designed to ensure comparable safety and efficacy of these antithrombotic agents, particularly in complex cardiac populations (50, 54).

Currently, there are insufficient outcome data to recommend DOACs in a patient with a Fontan circulation (50, 55). Important considerations regarding the prescription of a suitable thromboprophylactic agent are outlined in Table 1.

**TABLE 1:** Comparison of considerations when prescribing warfarin, aspirin, and direct oral anticoagulants.

	Warfarin	Aspirin	Direct oral anticoagulants
Mechanism of action	• Reduces synthesis of active Vitamin K dependent clotting factors	• Inhibits platelet aggregation	• Direct inhibition of factor Xa or thrombin
Considerations	• Affected by patient-related factors (e.g., medications, diet) • Increased risk of bleeding • Can be reversed by giving vitamin K (important if emergency surgery required) • Quality of Life may be decreased • Potential reduction in bone density	• Increased risk of bleeding • Not reversible • Aspirin resistance	• Increased risk of bleeding • Depend on renal and hepatic function for adequate clearance • Do not all have approved reversal agents • Insufficient outcome data to substantiate safety and efficacy in Fontan
Monitoring requirements	• Regular venous monitoring with INR (narrow therapeutic range)	• Not monitored	• Regular monitoring not required
Convenience	• Oral tablet • Regular monitoring required	• Oral tablet	• Oral tablet • Rapid offset, requires strict adherence for anticoagulative effect

*Modified from Attard et al. (10).*

### Aspirin and Warfarin

Fontan patients are generally prescribed lifelong thromboprophylactic treatment with aspirin or warfarin to mitigate the risk of thrombosis post-surgery. However, the superiority of aspirin or warfarin as a thromboprophylactic agent following Fontan palliation remains to be determined. Selection of a suitable thromboprophylactic regime must therefore consider mechanistic differences, drug interactions and monitoring requirement, as well as patient-related factors such as diet, concomitant medications and pre-existing conditions or history.

Warfarin is an oral anticoagulant that disrupts the vitamin K cycle, inhibiting the synthesis and activation of vitamin K-dependent clotting factors II, VII, IX, and X, as well as the anticoagulant proteins C, S, and Z. As a vitamin k antagonist (VKA), warfarin reduces the overall activation and efficacy of the coagulation cascade, permitting an antithrombotic state in the Fontan circulation. Warfarin use has a number of limitations, including a narrow therapeutic range, significant drug and food interactions and necessitating regular monitoring to ensure safe, controlled and effective anticoagulation (56).

Aspirin, or acetylsalicylic acid (ASA), is an oral antiplatelet drug that prevents the generation of thromboxane A2, irreversibly inhibiting platelet activation and aggregation to impair downstream platelet plug and thrombus formation (57). Although, monitoring of aspirin is not routinely performed in most clinical settings, the possible development of aspirin resistance and a consequential increased risk of thrombosis in a subset of the Fontan population must be considered. Preliminary estimates from small sample cohort studies of postoperative aspirin resistance in the Fontan population have ranged from 50 to 73% (58–60). However, future studies are needed to comprehensively assess the incidence of aspirin resistance in the Fontan patients, as well as its significance as a risk factor for thromboembolism.

With differing modes of action and considerations presented by each drug, several studies have aimed to compare outcomes of aspirin and warfarin after Fontan surgery. The study by Monagle et al. remains the only randomized trial to directly compare outcomes of warfarin and aspirin as thromboprophylaxis post-Fontan surgery (41). The initial results from a cohort of 111 Fontan patients, show that there was no significant difference in thrombotic outcomes between those receiving aspirin and warfarin over the two years post-Fontan surgery (41). However, a secondary analysis of this RCT revealed a 3.5-fold increased risk of thrombosis associated with poorly controlled warfarin therapy (≤30% of INR values within target range when compared to patients who consistently achieved target INR levels or received aspirin (31). This finding is consistent with a retrospective study from Faircloth et al. where high time within therapeutic INR for warfarin was associated with significantly lower rates of thrombotic and bleeding events in the Fontan population (61), indicating the importance of maintaining INR within a target range for effective warfarin thromboprophylaxis in the Fontan population.

A recent multicenter study by Attard et al. (3) utilized MRI, bone densitometry (dual-energy x-ray absorptiometry, DXA), bleeding and Quality of Life (QoL) tools to assess outcomes of aspirin and warfarin use after more than five years after Fontan surgery. This cross-sectional study provides some of the most comprehensive evidence of the long-term outcomes of thromboprophylaxis post Fontan surgery. The key findings of the study were the widespread prevalence of asymptomatic stroke (39%) and cerebral micro-hemorrhage (96%) in the Fontan population, irrespective of thromboprophylaxis type. In addition, high bleeding rates were reported in both groups, with bleeding being more frequent in the warfarin group. Bone mineral density (BMD) was reduced across the cohort compared with the general population; however, BMD was poorer in those receiving warfarin. In the warfarinized patient a reduction in BMD is most likely caused via warfarin’s inhibition of the vitamin K-dependent protein osteocalcin, impairing its key role in bone mineralization. Given the young age of the study cohort, inadequate bone mass accumulation is of particular concern as it is associated with increased fracture risk and osteoporosis in later life (62). Given the widespread levels of reduced BMD that were observed, BMD screening in the Fontan population, particularly those receiving long-term warfarin therapy may be warranted. Furthermore, selection of aspirin as primary thromboprophylaxis in the absence of contraindications may therefore improve bone health outcomes and in turn decrease a possible increased risk of fracture and osteoporosis in the Fontan population.

QoL has also become an increasingly significant metric used when assessing the wider impact of different clinical interventions in pediatric anticoagulant populations (63). Interestingly, in the Attard study, quality of life was similar between the warfarin and aspirin groups (3). Perhaps not surprisingly, in the warfarin group, home INR monitoring was associated with improved QoL scores compared to those receiving community monitoring. Previous studies have also demonstrated an improved QoL score when patients on warfarin commenced Home INR monitoring (64). Furthermore, a reduction in financial burden were demonstrated to both the family and the broader healthcare system when home INR monitoring was employed (65).

These findings, alongside the persistent debate regarding the relative efficacy of aspirin and warfarin, highlight that more favorable thromboprophylactic therapies are likely needed to resolve the significant residual risk of thrombosis and cerebrovascular injury in the aging Fontan population.

### Emerging Antithrombotic Therapies

Given the limitations of VKAs as thromboprophylaxis, there has been extensive research into novel therapies in the broader population indicated for anticoagulation, though studies specific to safety and efficacy in patients with a Fontan-class circulation remain in their infancy (50, 66). Among the new classes of oral anticoagulants that have been developed are direct thrombin inhibitors (dabigatran) and direct factor Xa inhibitors (apixaban, endoxaban, rivaroxaban). These novel antithrombotic agents are collectively referred to as direct oral anticoagulants, or DOACs.

Rivaroxaban, like apixaban and endoxaban, is a highly selective direct inhibitor of factor Xa (67). By acting primarily to inhibit FXa, rivaroxaban hinders the flux through the intrinsic and extrinsic pathways of the coagulation cascade, ultimately impairing downstream thrombin generation and thrombus formation (68). Dabigatran instead inhibits both fibrin-bound and unbound (free) thrombin directly, restricting its powerful thrombogenic function as the final effector in the coagulation cascade (69).

DOACs offer attractive benefits when compared to VKA anticoagulants such as warfarin, namely their rapid onset of action, minimal drug and food interactions and predictable pharmacokinetics, avoiding the need for regular patient monitoring (70). However, adequate renal function is crucial to avoid an increased risk of hemorrhage during DOAC use in the adult congenital heart disease (ACHD) population (71).

While evidence-based analysis of DOACs for primary thromboprophylaxis in the Fontan population is rare across the published literature, there is a growing body of research (66). In a single-center study, Georgekutty et al. reported the first retrospective data on the safety and efficacy of DOACs as either primary or secondary thromboprophylaxis in 21 adult patients with a Fontan-type circulation (72). Over a cumulative 316 patient-months, one thrombotic event occurred in a patient with a failing Fontan physiology and Protein-losing enteropathy, and no major bleeding events were observed (median follow-up 13 months).

A larger multicenter prospective study by Yang et al. assessed DOAC use in 530 ACHD patients, with 14% (*n* = 74) having a Fontan-class circulation (73). All major bleeds and 50% of thromboembolic events occurred in Fontan patients (*n* = 3, 4.1%), emphasizing the susceptibility of the Fontan population to hemostatic complications. Further analysis of this Fontan cohort nevertheless determined comparable rates of thromboembolism and major bleeding to VKAs as short-term thromboprophylaxis (74). A recent retrospective multicenter study by Kawamatsu et al. consisting of 139 adult Fontan patients similarly proposed that DOACs may present benefits over VKAs in safety and efficacy, reducing the risk of thrombosis and hemorrhage (75).

Although the safety and efficacy of DOACs compared to VKAs has primarily been evaluated in adults, a recent trial investigated the safety and efficacy of rivaroxaban compared to aspirin in children post-Fontan procedure (81). The UNIVERSE study compared thromboprophylaxis with rivaroxaban or aspirin in 112 children post-Fontan surgery and revealed that patients who received rivaroxaban had a similar safety profile to those who received aspirin. A lower thrombotic event rate was also observed in the rivaroxaban group (2% vs. 9%) however this difference was statistically insignificant (76).

However, recent literature has also suggested that DOACs may incur significant risks when used for thromboprophylaxis in circulations that are intercalated with artificial surfaces, as is the case for the Fontan population. Three independent trials assessing dabigatran or rivaroxaban against standard of care in cardiac surgery patients were terminated prematurely due to safety concerns, as there was an excess of thromboembolic and hemorrhagic events experienced by patients in the DOAC treatment groups (77–79).

These adverse trial events occurred in patients whose circulations were exposed to significant amounts of synthetic material—namely mechanical heart valves (77), Left Ventricular Assist Devices (78) and transcatheter aortic-valve replacements (79). Given the similar reliance on thrombogenic artificial surfaces in the two dominant current Fontan procedures, the lateral tunnel and extracardiac conduit (12, 32), these studies indicate the need for careful consideration of possible complications when assessing the use of DOACs in the Fontan circulation.

There are currently three ongoing studies examining the safety and efficacy of DOACs as thromboprophylaxis for adult congenital heart disease (ACHD). A prospective multi-center observational study is aiming to assess routine apixaban use in ACHD, including Fontan-class circulations, and atrial arrythmias (NCT03854149) (80). There is also one ongoing interventional randomized control trial evaluating the safety and efficacy of DOACs in the pediatric Fontan population. The SAXOPHONE study (Safety of ApiXaban On Pediatric Heart disease On the preventioN of Embolism), entails a comparison of antithrombotic viability and QoL between apixaban and existing standard of care anticoagulants—VKAs and low molecular weight heparin in acquired and congenital heart disease pediatric patients (NCT02981472) (54). The SAXOPHONE study will stratify patients based on diagnosis into three groups: single ventricle, acquired and all other congenital heart disease. The single ventricle group will include Fontan patients and therefore this group can be analyzed to determine the efficacy of apixaban in the pediatric Fontan population. The outcomes of these trials will be extremely important to the field.

## Conclusion

Recent literature has determined that cerebrovascular injury is a frequent occurrence after Fontan surgery, regardless of thromboprophylaxis type. In addition to TE incidence, several important secondary clinical outcomes have been identified that should be considered when determining an individual’s thromboprophylaxis regime.

Hence, the following recommendations should be considered for managing post-Fontan TE risk:

1. Where no further clinical complexities exist, it is reasonable to offer aspirin to Fontan patients as thromboprophylaxis. However, before one could be definitive about optimal thromboprophylaxis, consideration must bex given to important clinical features such as cardiac and lung function.
2. Routine BMD screening should be considered all Fontan patients, particularly those on long-term warfarin therapy. As with all populations that are particularly vulnerable to osteopenia/osteoporosis, vitamin D sufficiency and adequate dietary calcium intake are important factors to ensure bone mass accrual and maintenance. Furthermore, patients should be encouraged to engage in weight bearing exercise, in line with their physical capabilities.
3. When warfarin is indicated, a comprehensive anticoagulation service that facilitates home INR monitoring can be of great benefit to the patient in terms of both QoL and financially.
4. The routine use of DOACs as primary thromboprophylaxis after Fontan surgery is not currently recommended given the current limited evidence of efficacy in this cohort.

## Data Availability Statement

The original contributions presented in the study are included in the article/supplementary material, further inquiries can be directed to the corresponding author/s.

## Author Contributions

SV and CS: literature search. CA, SV, CS, VI, and PM: drafting of the manuscript. CA, VI, and PM: editing of the manuscript. All authors contributed to the article and approved the submitted version.

## Conflict of Interest

The authors declare that the research was conducted in the absence of any commercial or financial relationships that could be construed as a potential conflict of interest.

## Publisher’s Note

All claims expressed in this article are solely those of the authors and do not necessarily represent those of their affiliated organizations, or those of the publisher, the editors and the reviewers. Any product that may be evaluated in this article, or claim that may be made by its manufacturer, is not guaranteed or endorsed by the publisher.
